# Investigation of adverse reactions in healthcare personnel working in Level 3 barrier protection PPE to treat COVID-19

**DOI:** 10.1136/postgradmedj-2020-137854

**Published:** 2020-06-18

**Authors:** Niu Yuan, Wei-Xia Yang, Jian-Li Lu, Zhang-Hong Lv

**Affiliations:** Department of Respiratory Medicine, The First Affiliated Hospital of Zhejiang University School of Medicine, Hangzhou, China; Department of Respiratory and Gastroenterology, The First Affiliated Hospital of Zhejiang University School of Medicine, Hangzhou, China; Deparment of Orthopedics, The First Affiliated Hospital of Zhejiang University School of Medicine, Hangzhou, China; Department of Respiratory Medicine, The First Affiliated Hospital of Zhejiang University School of Medicine, Hangzhou, China

**Keywords:** RESPIRATORY MEDICINE (see Thoracic Medicine)

## Abstract

**Purpose of the study:**

The aim of our study was to investigate potential adverse reactions in healthcare professionals working in Level 3 barrier protection personal protective equipment (L3PPE) to treat patients with COVID-19.

**Study design:**

By using a convenience sampling approach, 129 out of 205 randomly selected healthcare professionals from the First Affiliated Hospital of Zhejiang University School of Medicine were invited to take part in a WeChat messaging app survey, Questionnaire Star, via a survey link. Healthcare personnel details were collected, including profession, years of professional experience and adverse reactions while wearing L3PPE. Survey results were divided by profession and years of professional experience; differences in adverse reactions were compared.

**Results:**

Among the 129 healthcare professionals surveyed, 21 (16.28%) were doctors and 108 (83.72%) were nurses. A total of 122 (94.57%) healthcare professionals experienced discomfort while wearing L3PPE to treat patients with COVID-19. The main reasons for adverse reactions and discomfort include varying degrees of adverse skin reactions, respiratory difficulties, heat stress, dizziness and nausea. Doctors had a lower incidence of rashes (χ^2^=4.519, p=0.034) and dizziness (χ^2^=4.123, p=0.042) when compared with nurses. Junior (8.5 years of experience or fewer) healthcare personnel also experienced a higher rate of heat stress when compared with senior personnel (more than 8.5 years greater) (χ^2^=5.228, p=0.022).

**Conclusion:**

More attention should be offered to healthcare personnel wearing L3PPE to treat patients with COVID-19 because they are susceptible to developing adverse reactions.

## INTRODUCTION

As of April 4, 2020, there have been 82 511 confirmed cases of the novel Coronavirus Disease 2019 (COVID-19) in China, following the first report of an unidentified patient with pneumonia on December 8, 2019. This has further evolved into a global pandemic, where six other countries (USA, Italy, Spain, Germany, France and Iran) have each reported over 50 000 confirmed cases of COVID-19 (as of April 4, 2020).^1^ At the initiation of the COVID-19 outbreak, the National Health Commission of the People’s Republic of China (NHCPRC) led all medical institutions in China to provide voluntary support in Hubei, which was the epicentre of the epidemic. The author took part in this volunteer support and went to Hubei, Wuhan, China, on February 17, 2020. During the 1 week of volunteer service while wearing level 3 barrier protection PPE (L3PPE), the author experienced discomfort including heat stress, perspiration, respiratory difficulties, facial skin indentation, nausea and vomiting. Furthermore, because respiratory difficulty is the main clinical manifestation of COVID-19,^2^ the author also experienced unprecedented emotional tension and stress.

Through interaction with fellow healthcare professionals, respiratory difficulty was found to be the most prevalent problem for medical personnel while wearing L3PPE. This discomfort gradually alleviated after the PPE was removed. The Technique Standard for Isolation in Hospitals (WS_T_311_2009),^3^ released on April 1, 2009 by the NHCPRC and implemented on December 1, 2009, requires the use of PPE for medical personnel during the treatment and nursing of infectious diseases, such as COVID-19. This involves wearing L3PPE that includes a disposable medical N95 protective mask, goggles, triple layers of medical gloves, a protective face shield, an isolation gown and medical protective clothing.

During the outbreak of the Severe Acute Respiratory Syndrome, a study from Singapore found that many medical personnel who wore PPE to work developed adverse skin reactions, such as dry skin, facial itch, acne and rash.^4^ After the outbreak of COVID-19, the Chinese Society of Dermatology studied 330 medical personnel from the Fever Clinic and Observation Ward for adverse skin reactions and found that 70% (234) of them developed skin barrier injuries with symptoms, such as skin burns, itch and pruritus.^5^ Apart from developing skin-related discomforts, medical personnel, when wearing PPE to work, can also exhibit respiratory discomforts. Person *et al* demonstrated that healthy individuals who wore surgical masks to undergo a 6-minute walk exercise exhibited significant respiratory difficulties.^6^ While there is insufficient evidence to suggest the development of dizziness and nausea in healthcare professionals when wearing PPE, we further included the investigation of digestive discomforts in this study due to the personal experiences of the author who experienced nervous and digestive discomforts after wearing a L3PPE to treat patients with COVID-19.

This study seeks to understand the potential skin, respiratory, nervous and digestive reactions in Chinese healthcare professionals who wear L3PPE to combat COVID-19. It also seeks to serve as a professional health advisory for other front-line healthcare professionals globally who are working to treat patients with COVID-19.

## METHODS

In January 2020, doctors, nurses, hospital infection prevention and control staff, and logistics personnel formed a front-line medical team under the directive of the NHCPRC. The medical team received formal PPE training from hospital infection prevention and control experts before treating patients with COVID-19.

In this study, we sent our survey link to our WeChat working group consist of 205 front-line healthcare professionals (doctors and nurses) from the First Affiliated Hospital of Zhejiang University School of Medicine and received 129 responses, thus making the response rate of 62.93%. They worked for an average of 4 hours daily while wearing PPE consisting of a disposable medical N95 protective mask, goggles, triple layers of medical gloves, a protective face mask, an isolation gown and medical protective clothing.

In order to prevent any risks of cross-contamination by the distribution of paper questionnaires, this study adopted a convenient sampling method approach that used a WeChat (messaging app) survey mini-programme. A survey invitation link was sent by Questionnaire Star (Changsha Ranxing Information Technology), to 129 random healthcare personnel to investigate potential skin, respiratory, nervous and digestive reactions while wearing L3PPE to treat patients with COVID-19. Details collected in the survey included profession, years of profession, rash, contact or allergic dermatitis, respiratory difficulties, heat stress, dizziness and nausea.

Survey respondents were divided into two groups based on their profession (doctors and nurses) and then were also divided into two groups based on a median length of professional experience of 8.5 years. Healthcare personnel with a fewer than <8.5 years of professional experience were considered to be ‘junior’ and those who had greater than 8.5 years of professional experience were considered to be ‘senior’. Adverse reactions were compared between the groups.

### Statistical methods

Adverse reactions between the junior and senior groups were considered as count data and comparisons of differences between the two groups were analysed using χ^2^ analysis, where p<0.05 is statistically significant. Statistical package, SPSS 22.0 (IBM, New York, USA) was used to perform the statistical analysis.

## RESULTS

A total of 129 healthcare professional involved in the treatment of COVID-19 were included in this study, comprising 21 doctors (16.3%) and 108 nurses (83.7%). The average length of professional experience was 9.40±5.56 years, whereas the median length of professional experience is 8.5 years. Among them, the distribution of length of profession experience <8.5 years (junior) and ≥8.5 years (senior) was 50 and 79, respectively.

The overall adverse reaction rate for all healthcare personnel was 94.6%, whereas the adverse reaction rate for doctors and nurses was 81.0% and 97.2%, respectively (χ^2^=7.118, p=0.008). The overall adverse reaction rate for junior healthcare professionals was 96.0%, whereas the overall adverse reaction rate for senior healthcare professionals was 93.7% (χ^2^=0.324, p=0.569). We found that 61.9%, 4.8%, 14.3%, 38.1%, 57.1%, 28.6% and 14.3% experienced facial skin indentation, rashes, dermatitis, respiratory difficulties, heat stress, dizziness and nausea, respectively, in doctors, whereas, 74.1%, 25.9%, 25.0%, 59.3%, 70.4%, 52.8% and 25.9% developed facial skin indentation, rashes, dermatitis, respiratory difficulties, heat stress, dizziness and nausea, respectively, in nurses (table 1). We also found that 74.0%, 24.0%, 46.7%, 62.0%, 80.0%, 44.0% and 24.0% experienced facial skin indentation, rashes, dermatitis, respiratory difficulties, heat stress, dizziness and nausea, respectively, in junior healthcare professionals, whereas, 70.9%, 21.5%, 20.3%, 51.9%, 60.8%, 51.9% and 24.1% developed facial skin indentation, rashes, dermatitis, respiratory difficulties, heat stress, dizziness and nausea, respectively, in senior healthcare professional. The biggest discrepancies in adverse reaction rate appeared to be between doctors and nurses. Significantly more nurses were potential to the rashes (25.9% vs 4.8%, χ^2^=4.519, p=0.034) and dizziness (52.8% vs 28.6%, χ=4.123, p=0.042) when compared with doctors. Moreover, the incidence of heat stress was notably higher in juniors than in seniors (80.0% vs 60.8%, χ^2^=5.228, p=0.022).

**Table 1:** Results from our survey assessing adverse reactions in healthcare staff working in Level 3 barrier protection personal protective equipment to treat COVID-19.

	Survey assessing adverse reactions in healthcare staff working in L3PPE to treat COVID-19			
	Doctors (n=21)	Nurses (n=108)	Juniors (n=50)	Seniors (n=79)
Facial skin indentation	13 (61.9%)	80 (74.1%)	37 (74.0%)	56 (70.9%)
Rash	1 (4.8%)	28 (25.9%)	12 (24.0%)	17 (21.5%)
Dermatitis	3 (14.3%)	27 (25.0%)	14 (46.7%)	16 (20.3%)
Respiratory difficulties	8 (38.1%)	64 (59.3%)	31 (62.0%)	41 (51.9%)
Heat stress	12 (57.1%)	76 (70.4%)	40 (80.0%)	48 (60.8%)
Dizziness	6 (28.6%)	57 (52.8%)	22 (44.0%)	41 (51.9%)
Nausea	3 (14.3%)	28 (25.9%)	12 (24.0%)	19 (24.1%)

## DISCUSSION

The outbreak of COVID-19 initiated in China, Hubei, Wuhan, and rapidly spread across the rest of the country in a mere period of 30 days.^7^ The major transmission route of COVID-19 is transmission via respiratory droplets and through close contact as well as the potential of spreading via aerosol transmission.^8^ Implementations of infection prevention and control can effectively limit the transmission of the virus and is of utmost importance to the personal safety of healthcare personnel.^9^ However, as of February 24, 2020, the NHCPRC reported at the WHO-China Joint Expert Inspection Team Press Conference that there were 3387 confirmed cases of healthcare personnel infected with COVID-19. This was analysed by the director of the National Hospital Infection Management and Quality Control Center and attributed to the poor understanding of the disease and limited self-protection awareness of healthcare personnel during the onset of the outbreak.^10^ Following the outbreak of COVID-19, the NHCPRC organised and lead 42 000 core healthcare personnel to provide medical support in Wuhan and various parts of Hubei. As of March 20, 2020, due to the effective and appropriate usage of PPE, none of the 42 000 healthcare personnel were infected and about 12 000 of them have returned home after successfully accomplishing their support mission.

While the correct usage of PPE is the most important approach to protect healthcare personnel from potential risks of the deadly viral infection, there is an immediate need to improve the design and utility of PPE and further ensure the well-being of healthcare personnel.^11^ A randomised control study reported relevant backpain related to carrying a respirator as well as fluid loss while working at 28°C as being major limiting factors of wearing the medical protective suit and thereby affecting the usage of PPE.^12^ The fully enclosed design of current PPE worn by healthcare personnel greatly reduces its comfort and usability. Verbeek JH *et al* indicated in a systematic review that a report, with weak evidential support, claimed that using a breathable PPE could still effectively prevent infections while allowing increased usability and comfort.^13^ However, this must be further validated with additional safety tests. At present, most studies that investigated the comfort and usability of PPE have generally reported adverse reactions of the skin mucosa.^14^ In this comprehensive study, our results indicate the incidence of overall adverse reactions in healthcare personnel to be 94.57%. This predominantly includes varying degrees of facial skin indentation, respiratory difficulties, heat stress, dizziness and nausea. Comparisons between the groupings indicate the incidence of rashes and dizziness to be lower in doctors than in nurses, whereas the occurrence of heat stress is notably higher in junior than in senior healthcare personnel.

Our study discovered that the high rates of adverse reactions experienced by healthcare personnel due to the usage of L3PPE in treatment of COVID-19 not only include previously reported skin mucosa discomfort reactions but include multiple adverse reactions linked to the respiratory, nervous and digestive systems. Based on our literature review of related publications in PubMed, Web of Science and the China National Knowledge Infrastructure, we believe this is the first study that comprehensively uncovers the potential adverse reactions with the usage of PPE in an event of a major public health emergency. It is also important to point out that the results of this study are primarily based on self-evaluation of surveyed personnel and have not been clinically validated. This study was limited in our means to grade and evaluate various reported adverse reactions. Moreover, our study result was based on a single centre and received partial responses that might not be representative of all healthcare personnel working in L3PPE to treat COVID-19. Nonetheless, we believe that the results presented in this study could offer some useful professional health insights to support fellow global medical compatriots who are battling on the front line to contain the COVID-19 viral outbreak.

**Main messages**

- Almost all the healthcare professionals experienced discomfort while wearing L3PPE (Level 3 barrier protection personal protective equipment) to treat patients with COVID-19. It is important for healthcare personnel to be well prepared in the work to treat patients with COVID-19.
- The main reasons for adverse reactions and discomfort while wearing L3PPE include varying degrees of adverse skin reactions, respiratory difficulties, heat stress, dizziness and nausea.
- The significant difference in rash rates between doctors and nurses working in L3PPE was presented.
- The significant difference in heat stress rate between junior and senior healthcare professionals working in L3PPE was presented.

**Current research questions**

- Would healthcare professionals working in L3PPE experience varying degrees of adverse reactions, including skin reactions, respiratory difficulties, heat stress, dizziness and nausea?
- Is there a significant difference in adverse reactions between doctors and nurses working in L3PPE?
- Is there a significant difference in adverse reactions between junior and senior healthcare professionals working in L3PPE?

**What is already known on the subject**

- The overall adverse reaction rate for all healthcare personnel wearing L3PPE was 94.57%.
- The overall adverse reaction rate and rash rates between doctors and nurses showed significant difference.
- The heat stress rate between junior and senior healthcare professionals also showed significant difference.
